# A Rare Cause of Colon Perforation After Percutaneous Nephrolithotomy—A Case Report and Review of the Literature

**DOI:** 10.1155/2024/4475216

**Published:** 2024-11-14

**Authors:** Christina S. Boutros, Alexander W. Loftus, Aria Bassiri, Laura E. Davis, Randy Vince, Jillian Sinopoli, Leonidas Tapias, Philip A. Linden, Christopher W. Towe, Boxiang Jiang

**Affiliations:** ^1^Department of Surgery, Division of Thoracic and Esophageal Surgery, University Hospitals Cleveland Medical Center, Cleveland, Ohio, USA; ^2^Department of Urology, University Hospitals Cleveland Medical Center, Cleveland, Ohio, USA

## Abstract

Staghorn calculi pose challenges in urology, often necessitating more invasive procedures such as percutaneous nephrolithotomy (PCNL) to clear a large stone burden with fewer procedures. Here we present a case of a 76-year-old female with chronic kidney disease and a malrotated right pelvic kidney who underwent PCNL for a 3.5 cm staghorn calculus. Postoperatively, she developed a rare complication of ascending colon perforation, requiring emergent surgical intervention including exploratory laparotomy and right hemicolectomy. Colon perforation during PCNL is rare (0.3%−0.8%). Preoperative imaging, namely computed tomography (CT) of the abdomen and pelvis, is crucial to identify anatomical variations and mitigate the risk of injury. Conservative management strategies have demonstrated success in similar cases, emphasizing the importance of prompt recognition and multidisciplinary management. This case contributes to the paucity of literature regarding this rare complication underscoring the necessity for detailed preoperative planning to avoid complications in PCNL, especially in patients with complex renal anatomy. Future research should focus on developing tailored guidelines for PCNL in patients with anatomical abnormalities to enhance procedural safety and optimize outcomes.

## 1. Introduction

Staghorn calculi, named for their antler-like shape, represent a challenging clinical entity in urology, often occupying a large volume within the collecting system of the kidney [1, 2]. These calculi are typically composed of struvite and can lead to recurrent infections, obstruction, and ultimately, renal failure if not effectively managed [3, 4]. Conventional management strategies include staged ureteroscopy and percutaneous nephrolithotomy (PCNL), with the latter being particularly favored in cases involving large or complex stone burdens [5, 6].

Further complicating the management of staghorn calculi are anatomical anomalies such as a malrotated or ectopic kidney [7–9]. The presence of a low lying, malrotated right pelvic kidney, as seen in the patient discussed in this case report, poses significant surgical challenges due to abnormal orientation and altered vascular and collecting system anatomy [9]. These anatomical variations necessitate careful procedural planning and execution to avoid complications.

Percutaneous access is a crucial technique in the management of obstructive uropathy secondary to staghorn calculi, providing entry to the renal collecting system for both diagnostic and therapeutic procedures [10]. However, obtaining percutaneous renal access is not devoid of risks, particularly in kidneys with variant anatomy. This case report focuses on colon perforation following PCNL, an unusual and serious complication, in a patient with a staghorn calculus and a malrotated pelvic kidney. We review this rare complication within the context of current literature to enhance understanding and improve the management of future cases.

## 2. Case Report

**Figure 1:**
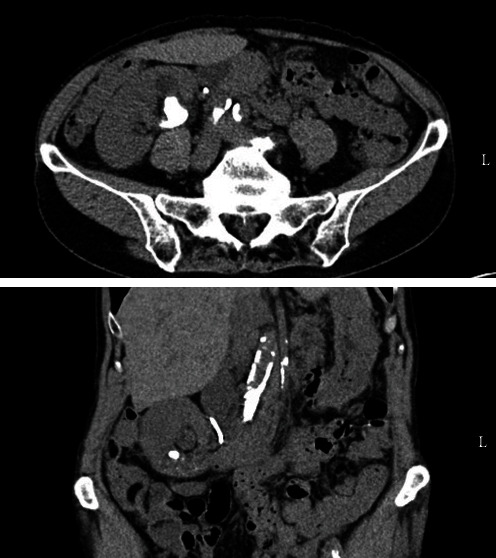
Representative images of CT scan of the abdomen and pelvis showing proximity of bowel to the right kidney. CT, computed tomography.

Here we present a case of a 76-year-old female with a past medical history of chronic kidney disease, tularemia, hypertension, and nephrolithiasis who presented with 3.5 cm staghorn caliculi in the right renal pelvis and lower pole, mild right-sided hydronephrosis, and low lying malrotated right pelvic kidney, which was noted on preoperative computed tomography (CT) imaging (Figure 1). She previously underwent ureteroscopy which failed to eliminate the stone due to size. Right double-J ureteric stent was placed at the time of ureteroscopy for renal decompression. Patient preoperative imaging was reviewed between the time of ureteroscopy and subsequent PCNL for surgical planning.

Patient was scheduled for right-sided PCNL with planned access through the upper pole via fluoroscopic biplanar approach. On the day of the procedure, upper pole access was planned; however, due to large stone burden, a ureteroscope was unable to be advanced past the stone into the desired upper pole location to obtain end-to-end access. Lower pole access was therefore obtained directly onto the lower pole stone under biplanar fluoroscopic guidance. Specifically, images were obtained via c-arm straight on with patient in a prone position as well as with the c-arm obliqued 30° toward the surgeon. An 18-gauge access needle was then adjusted until the trajectory aligned with the target (typically, the distal end of the ureteroscope, but in this case, the lower pole stone itself) at roughly a 60° angle from the desired puncture site. A breath hold was initiated by anesthesia, and the needle was advanced under fluoroscopic guidance onto the lower pole stone with care taken to ensure this insertion site was below the 12th rib on palpation. Stylet was removed and proper intracalyceal positioning was confirmed by immediate return of urine. A guidewire was passed through the access needle, which was subsequently removed, the percutaneous tract was dilated in the standard fashion, and a 24 French (Fr) access sheath was inserted. Upon entry into the collecting system, a large lower pole stone was identified, and lithotripsy was performed using the Trilogy lithotripter. Retrograde ureteroscopic access was maintained simultaneously and a ureteroscope was used to confirm stone clearance in the renal collecting system and the right ureter. At the end of the case, a few small stones remained in a lower pole diverticula. Clearance of these stones was attempted by ureteroscope, flexible cystoscope, and rigid nephroscope without success. A stent was, therefore, placed with a planned return for a staged ureteroscopy. An antegrade nephrostogram was performed at the conclusion of the case which revealed no extravasation of contrast. The patient was discharged home the same day.

On postoperative day 2, she presented to the emergency department with fatigue, lightheadedness, and abdominal pain. Clinically she was noted to be ill appearing and was found to have new-onset atrial fibrillation, hypotension, and lactic acidosis concerning for septic shock. She was resuscitated and started on broad-spectrum antibiotics. CT scan of the chest, abdomen, and pelvis was obtained which revealed pneumomediastinum and a right pleural effusion as well as pneumoperitoneum and gas and fluid tracking along the right retroperitoneal space into the chest concerning for perforated viscus. Patient was brought emergently to the operating room for exploratory laparotomy and possible right pleural exploration. She was found to have ascending colon perforation into the retroperitoneum with purulent and feculent contamination noted. She underwent right hemicolectomy, right retroperitoneal debridement, and temporary wound vac closure. A right chest tube was also placed with drainage of 2 L of serous fluid. The pleural fluid was not purulent and judged to be most likely reactive in nature; therefore, chest exploration was deferred. Postoperatively, the patient was clinically improved. She was taken back to the operating room for an end ileostomy creation, colonic mucus fistula, and abdominal closure two after exploratory laparotomy and was eventually the discharged to a skilled nursing facility on postoperative day 15 with an otherwise uncomplicated course.

## 3. Discussion

Colon perforation following PCNL is a rare but serious complication. Whereas major complications such as significant hemorrhage, injury to organs such as the liver, spleen, or colon are reported to occur at rates of 1%–3% [11], the rate of colon perforation ranges from 0.3% to 0.8% in literature. Risk factors for colon perforation include thin body habitus, horseshoe kidney, retrorenal colon, history of prior intestinal surgeries, and using the posterior axillary line for access [11]. The anatomical anomalies present in this case, notably the low position and malrotation of the kidney, placed this patient at significantly greater risk for colon injury due to deviation from the typical anatomical landmarks used in guiding such procedures. In the normal anatomical setting, the colon is at a relatively low risk of injury during PCNL due to its anterior position to the kidneys. However, in the case of a pelvic kidney, especially one that is malrotated, the colon may lie in close proximity to or even posterior and lateral to the renal collecting system [12]. This altered spatial relationship significantly elevates the risk of inadvertent colonic injury during PCNL.

A review of similar cases indicates that though colon perforation associated with PCNL is not commonly reported, when it occurs, it necessitates immediate recognition and management. A retrospective study of 1270 PCNL procedures conducted over 6 years identified colonic perforation in only 10 patients (0.8%). Conservative management, including repositioning of the nephrostomy tube to serve as a percutaneous colostomy, ureteral stent insertion, antibiotics, bowel rest, and total parenteral nutrition, resulted in successful healing of the colon in all cases in this series, highlighting the importance of early detection and prompt intervention in managing this complication [13]. Another series of 671 PCNL cases reported the incidence of colon perforation at 0.3% [14]. Although rare, some studies report that colonic perforation is more frequently observed during left-side procedures, lower calyceal punctures, in elderly individuals, and in patients with conditions like horseshoe kidneys or chronic colonic distension. Preoperative CT scanning is crucial to ensure that the retroperitoneal colon is not in the intended puncture path, thus preventing this serious complication [15, 16]. One study described an 18-year experience with PCNL based on a retrospective review of 5260 procedures. Among these cases, colonic perforation occurred in only 11 patients, predominantly affecting the right side (0.2%). Conservative management was successful in all cases, involving strategies such as nephrostomy tube repositioning, ureteral stent insertion, broad-spectrum antibiotics, bowel rest, and total parenteral nutrition, leading to complete healing without serious complications [17].

Pleural violation represents another rare complication of PCNL [18]. Though the 12th rib is often used as a landmark for the superior limit of possible access to avoid pleural injury, patient anatomy and location of stone burden sometimes necessitate supracostal access, particularly when access is obtained into the upper pole. Pleural injuries in PCNL tend to be more common in cases where upper pole access was obtained [19]; however, these injuries may still be seen in up to 4.5% of patients even with an infracostal approach as was the case in [20]. Such pleural breaches may cause injuries ranging from small violations that can be managed conservatively with observation alone to more severe sequelae such as severe hydropneumothorax requiring surgical interventions such as chest tube placement or thoracostomy [21].

Preventive measures are critical in reducing the risk of such complications. The utilization of advanced imaging techniques such as CT-guided access can help in accurately mapping the anatomy before the procedure [22]. Intraoperative ultrasound (US) guidance alone or in combination with fluoroscopy has also been utilized successfully to obtain percutaneous access during PCNL with comparable operative time and stone-free rate, decreased radiation, and improved visualization of structures between the puncture site and target anatomy including the viscera [23, 24].

Management of colon perforation following PCNL requires a multidisciplinary approach, involving urologists, radiologists, and general surgeons. The primary goal is sepsis control. In the reported series, conservative management of all colonic perforations was feasible in stable patients likely due to the retroperitoneal nature of these injuries [25]. In contrast to this case series, in our case, the patient presented in septic shock with evidence of pneumoperitoneum requiring prompt surgical exploration. Clearly, in this instance, conservative management was not an option as delaying surgical intervention could lead to serious morbidities and mortality of this patient.

This case adds to the limited literature regarding the severity of the colon perforation following PCNL. All perforations in the previous reported series were managed without surgical exploration and colonic resection. However, not all patients with colonic perforation after PCNL are able to be managed conservatively as is shown here. This case also reinforces the need for vigilance during PCNL in cases with atypical anatomy, with preparedness to recognize and swiftly manage serious complications. Preoperative evaluation in patients with variant renal anatomy should consist of CT of the abdomen and pelvis to recognize the anatomical relationships and risk factors for colon perforation. Lastly, our experience suggests a potential area for further research into best practices and guidelines specific to performing PCNL in patients with renal anomalies.

## 4. Conclusion

Patients with variant renal anatomy are at increased risk of colon perforation and pleural injury during PCNL. These patients should be evaluated preoperatively utilizing CT imaging to identify the safety of standard anatomic approaches. Perioperative percutaneous access can be optimized through the use of CT or US-guided access in challenging cases, particularly those with aberrant anatomy, to help mitigate risk of injury. We present a rare case which required emergent surgery due to complications and demonstrates the serious morbidity associated with unrecognized colonic perforation following PCNL in a patient with an ectopic, malrotated kidney. This case illustrates that due to altered anatomy, PCNL may not always be the optimal treatment option, and it should be considered only when other alternatives are not viable.

## Data Availability

The data that support the findings of this study are available from the corresponding author upon reasonable request.
